# Fingolimod Treatment in Relapsing-Remitting Multiple Sclerosis Patients: A Prospective Observational Multicenter Postmarketing Study

**DOI:** 10.1155/2015/763418

**Published:** 2015-07-22

**Authors:** Rocco Totaro, Caterina Di Carmine, Gianfranco Costantino, Roberta Fantozzi, Paolo Bellantonio, Aurora Fuiani, Ciro Mundi, Stefano Ruggieri, Carmine Marini, Antonio Carolei

**Affiliations:** ^1^Department of Neurology, University of L'Aquila, L'Aquila, Italy; ^2^Department of Neurology, Ospedali Riuniti, Foggia, Italy; ^3^Department of Neurology, IRCCS MEUROMED, Pozzilli, Italy

## Abstract

*Objective*. The aim of this prospective observational multicenter postmarketing study was to evaluate fingolimod efficacy in a real world clinical setting. *Methods*. One hundred forty-two subjects with relapsing-remitting multiple sclerosis (RRMS) were enrolled in three multiple sclerosis centers throughout Central and Southern Italy between January 2011 and September 2013. After enrollment, regular visits and EDSS assessment were scheduled every 3 months, and MRI scan was obtained every 12 months. Patients were followed up from 1 to 33 months (mean 14.95 ± 9.15 months). The main efficacy endpoints included the proportion of patients free from clinical relapses, from disability progression, from magnetic resonance imaging activity, and from any disease activity. *Results*. Out of 142 patients enrolled in the study, 88.1% were free from clinical relapse and 69.0% were free from disability progression; 68.5% of patients remained free from new or newly enlarging T2 lesions and 81.7% of patients were free from gadolinium enhancing lesions. Overall the proportion of patients free from any disease activity was 41.9%. *Conclusions*. Our data in a real world cohort are consistent with previous findings that yield convincing evidence for the efficacy of fingolimod in patients with RRMS.

## 1. Introduction

Multiple sclerosis is a chronic inflammatory disease in which autoreactive lymphocytes induce inflammatory damage to myelin sheaths of the central nervous system (CNS). The morphologic hallmarks of multiple sclerosis are demyelination, inflammation, axonal damage, and gliosis with loss of oligodendrocytes and neurons. Treatment options have widened in the last decade, leading to fingolimod (FTY270) approval as the first oral agent proved to be effective in reducing relapse rate and disability progression in relapsing remitting multiple sclerosis (RRMS). Fingolimod acts as a sphingosine-1-phosphate receptor (S1PR) modulator retaining pathogenic autoreactive T cells in the lymph nodes and preventing their infiltration into the CNS [1–4]. In addition, some studies suggest that fingolimod biological activity would be underpinned, to some extent, by its direct interaction with neural cells expressing S1PR [5, 6].

The efficacy of oral fingolimod was investigated in two large phase 3 clinical trials comparing oral fingolimod doses of 0.5 mg and 1.25 mg once daily in RRMS [7–10]. The FTY720 Research Evaluating Effects of Daily Oral Therapy in Multiple Sclerosis (FREEDOMS) trial showed significant efficacy of fingolimod in reducing the annualized relapse rate (ARR), the risk of disability progression, and the number of new or newly enlarging brain lesions on magnetic resonance imaging (MRI) [7, 8, 10]. The second pivotal trial (Trial Assessing Injectable Interferon versus FTY720 Oral in Relapsing-Remitting Multiple Sclerosis, TRANSFORMS) confirmed preliminary results, as fingolimod proved to be effective in reducing the ARR and MRI disease activity measures but failed to slow disability progression, compared to interferon beta 1-a [9].

In phase 2, phase 3, and extension studies, fingolimod exhibited a relatively good safety and tolerability profile. Most commonly reported adverse events in the fingolimod group were mild infections, mainly of the lower respiratory tract, increased alanine aminotransferase levels, bradycardia, first and second degree atrioventricular blocks at the time of first administration, hypertension, macular edema, mild decrease in lung function, and lowering of peripheral blood lymphocyte count, as expected from the mechanism of action of fingolimod [11–13].

The European Medicines Agency (EMA) approved fingolimod as a single agent disease modifying therapy in patients with unsatisfactory disease control despite treatment with a beta interferon or in treatment-naïve patients with rapidly evolving severe multiple sclerosis, providing a second-line treatment option in highly active RRMS. A recent observational population study showed similar efficacy of fingolimod compared with natalizumab on clinical measures, as defined by ARR and risk of disability progression, in an unselected RRMS cohort [14].

The aim of this prospective postmarketing multicenter observational study was to evaluate the clinical efficacy of fingolimod in RRMS patients in a real clinical practice setting.

## 2. Materials and Methods

### 2.1. Patients and Clinical Parameters

We report prospectively collected data from 142 patients diagnosed with RRMS according to McDonald criteria, in three multiple sclerosis centers throughout Central and Southern Italy, starting fingolimod treatment. Patients were consecutively enrolled between January 2011 and September 2013.

Baseline demographic and clinical data are reported in Table 1. Out of 142 patients, 95 (66.9%) were female; mean age was 39.56 ± 9.11 years (39.63 ± 8.15-year males, 39.53 ± 9.59-year females) with a mean disease duration of 11.37 ± 6.74 years (11.01 ± 6.13-year males, 10.62 ± 7.44-year females). Demographic characteristics did not differ significantly between groups.

Mean duration of the fingolimod treatment was 14.95 ± 9.15 months (range 1–33 months).

Thirty-four patients (23.94%) received fingolimod ≥ 24 months, 47 patients (33.09%) received fingolimod between 12 and 23 months, 33 patients (23.23%) received fingolimod between 6 and 11 months, and 28 patients (19.71%) received fingolimod between 1 and 5 months.

Patients were included in accordance with the Italian Drug Agency (AIFA) eligibility criteria for reimbursement of the drug. Criteria included patients on previous immunomodulant treatment for at least 12 months who had experienced at least one relapse in the last year with at least 9 T2 lesions at MRI or an increased lesion burden or at least one gadolinium-enhanced lesion or patients with severe multiple sclerosis with a fast progression/evolution, even if not previously treated with immunomodulant treatments, with ≥2 relapses with accumulation of disability during the last year, and with new T2- or gadolinium-enhanced lesions on MRI, compared with a previous MRI examination conducted during the previous 12 months.

Patients switching from natalizumab to fingolimod were also recruited and started treatment after a wash-out period of three months. All these patients discontinued natalizumab due to safety concerns for positive JCV status and overall treatment duration longer than 24 months, despite being clinically and radiologically stable under natalizumab treatment.

Patients underwent complete cardiac evaluation before starting fingolimod in order to exclude heart rate and atrioventricular conduction alterations. Moreover, a continuous electrocardiographic monitoring for six hours after the administration of the first dose was performed in all patients. Every hour, arterial pressure values were also registered.

Ophthalmologic evaluation with optical coherence tomography was performed at baseline, at 3 and 6 months and every 6 months thereafter, in order to monitor potential adverse events such as macular oedema.

Neurological examination and EDSS assessment were performed at baseline and during follow-up visits. EDSS was registered at baseline and every 6 months.

MRI scans were obtained at baseline, 12 and 24 months. Imaging data were collected at MRI facilities of the participating sites and reviewed by experienced neuroradiologists. MRI imaging was performed at 1.5 T and at least axial FLAIR, T2 weighted, and T1 gadolinium-enhanced sequences were obtained for all exams. MRI scans were analyzed for new or newly enlarging T2 lesions and gadolinium enhancing lesions.

Adverse events were recorded during the routine clinic visits or at their occurrence.

All patients were treated at a dose of fingolimod 0.5 mg once daily.

The study was approved by the Azienda Sanitaria Locale Avezzano-Sulmona-L'Aquila Ethics Committee and conducted in conformance with the ethical principles of the Declaration of Helsinki. Written informed consent was obtained from all patients.

### 2.2. Study Endpoints

The main end points were the proportion of patients free from clinical relapses; from confirmed disability progression, as defined by sustained increase of ≥1.0 point in the EDSS score (or ≥1.5 points if the baseline was <1.0) confirmed after 12 weeks; from new or newly enlarging T2 lesions; from gadolinium enhancing lesions; from any disease activity, as defined by the occurrence of relapse or confirmed disease progression or new disease activity at MRI.

ARR and EDSS score variations at 6, 12, 18, and 24 months with respect to basal values were also considered.

An intention to treat analysis was planned.

### 2.3. Statistical Analysis

Continuous variables were reported as means, standard deviations and range while discrete data were reported in contingency tables as absolute and relative frequencies.

The Mann-Whitney *U*-test or Wilcoxon tests were used where appropriate. The Cox multivariate model was used to test the influence of prognostic factors (age, sex, duration of the disease, number of previous treatments, EDSS score before fingolimod start, number of contrast-enhancing lesions at MRI before fingolimod start, previous immunosuppressant treatments, and previous natalizumab treatment).

The selected critical value for statistical significance was 0.05. Contingency tables were analyzed by *χ* ^2^ test where appropriate. Binary efficacy responses (yes/no, present/absent) were reported as absolute and relative frequencies. Furthermore, the appearance of events during the study was performed by the Kaplan-Meier (product-limit) survival analysis.

## 3. Results

**Figure 1:**
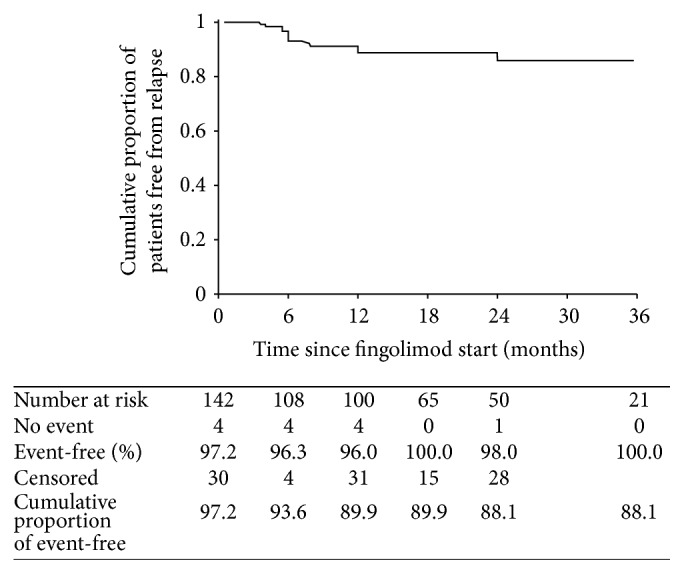
Kaplan-Meyer plot of cumulative proportion of patients remaining free from relapse over time from start of fingolimod therapy.

**Figure 2:**
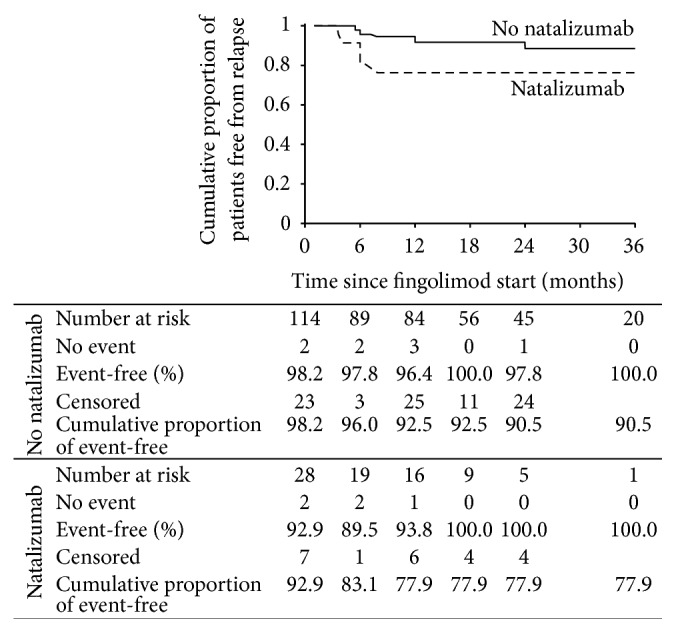
Kaplan-Meyer plot of cumulative proportion of patients remaining free from relapse by prior exposure to natalizumab over time from start of fingolimod therapy.

In the overall population, the cumulative proportion of patients free from clinical relapse was 88.1% (Figure 1).  Figure 2 shows the cumulative proportion of patients free from clinical relapse with respect to prior exposure to natalizumab. Patients without previous natalizumab exposure had improved relapse-free survival compared with those previously treated with natalizumab (90.5% versus 77.9%; *p* = 0.021).

Mean time to relapse was 29.6 months (95% confidence interval [CI], 28.025–31.202). Time to first relapse significantly decreased in patients receiving previous natalizumab treatment (23.767 ± 2.218 versus 30.411 ± 0.778; *p* = 0.021).

ARR significantly reduced after treatment with fingolimod. Values decreased from 1.14 to 0.14 at 6 months, to 0.11 at one year, and to 0.09 at 2 years of treatment (*p* < 0.0001). At 6 months of follow-up, ARR was significantly associated with the number of relapses in the year preceding the start of fingolimod treatment (*p* = 0.049) and with the previous use of natalizumab (*p* = 0.018). No associations were found at 12 and 24 months.

**Figure 3:**
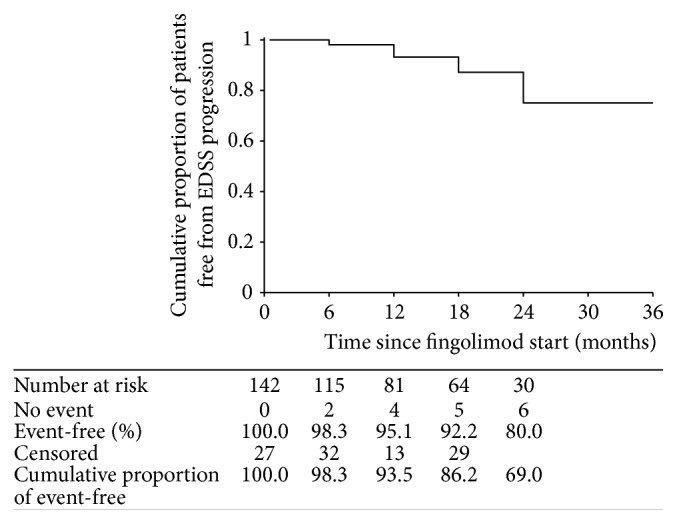
Kaplan-Meyer plot of cumulative proportion of patients remaining free from Expanded Disability Status Scale (EDSS) progression over time from start of fingolimod therapy.

As shown in Figure 3, the cumulative proportion of patients free from confirmed EDSS progression was 69.0%. During the study period, mean EDSS score decreased from 2.7 ± 1.1 at baseline to 2.5 ± 1.1 at 18 months (*p* = 0.13). EDSS evaluation at 24 months disclosed a trend toward further decrease, though sample size was too small to reach statistical significance (2.7 ± 1.1 versus 2.3 ± 0.9; *p* = 0.501). Mean time to confirmed EDSS progression was 28.6 months (95% CI, 26.9–30.3). Subgroup analysis failed to show any difference in time to disability progression for patients undergoing previous natalizumab treatment. Multivariate regression analysis showed no correlation between the risk of disability progression and age, gender, disease duration, number of previous treatments, previous immunosuppressant therapies, EDSS at baseline, previous contrast enhancing lesions, or previous natalizumab treatment.

MRI scans were obtained at baseline in all patients and in all 81 and 34 patients that reached 12 and 24 months of follow-up, respectively.

**Figure 4:**
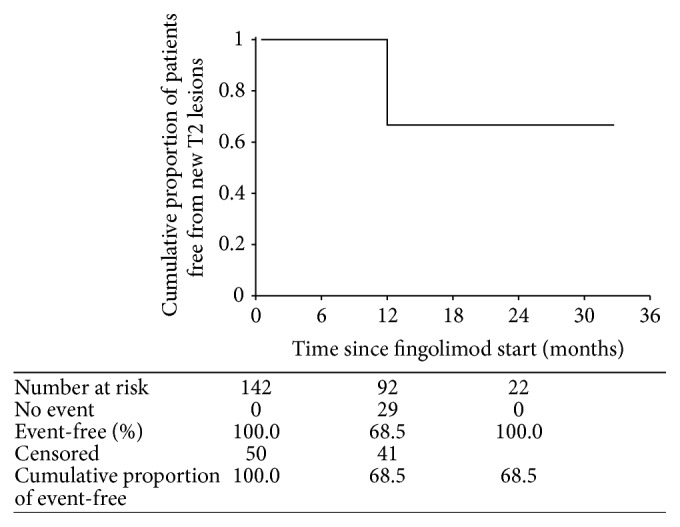
Kaplan-Meyer plot of cumulative proportion of patients remaining free from new or newly enlarging T2 lesions over time from start of fingolimod therapy.

As shown in Figure 4, 68.5% of patients were free from new or newly enlarging T2 lesions. Time to recurrence of new T2 lesions was 25.8 months, on average (95% CI, 23.8–27.9). Multivariate regression analysis found that EDSS score at baseline was the only predictor of new or newly enlarging T2 lesion occurrence, as EDSS score at baseline was significantly lower in patients with new T2 lesions on follow-up MRI (*p* = 0.016).

**Figure 5:**
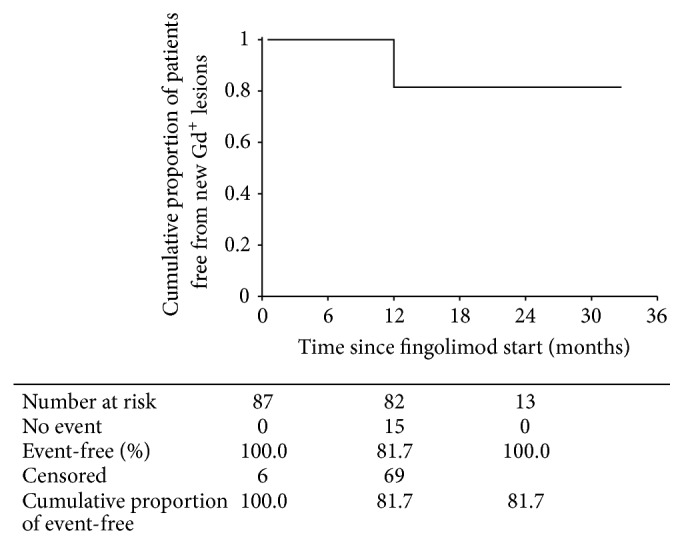
Kaplan-Meyer plot of cumulative proportion of patients remaining free from new gadolinium enhancing (Gd^+^) lesions at magnetic resonance imaging over time from start of fingolimod therapy.

Figure 5 shows that 81.7% of patients were free from new gadolinium enhancing lesions. Mean time to the appearance of a new enhancing lesion was 28.9 months (95% CI, 27.2–30.7). New contrast enhancing lesions were more common in patients undergoing previous natalizumab treatment (26.7% versus 15.3%, *p* = 0.046).

**Figure 6:**
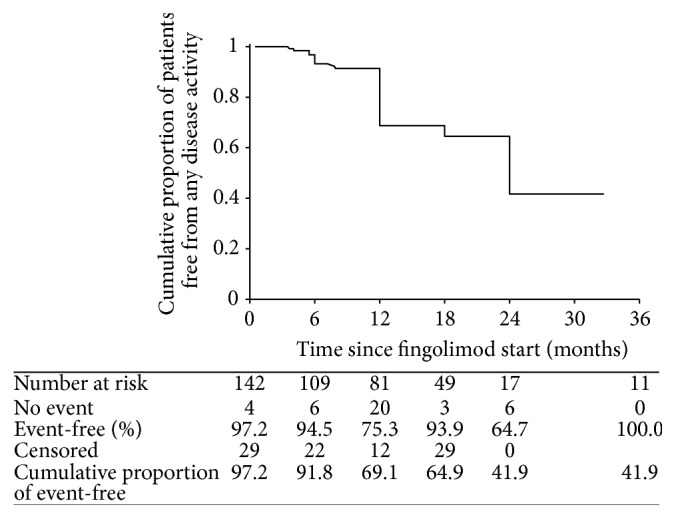
Kaplan-Meyer plot of cumulative proportion of patients remaining free from any disease activity over time from start of fingolimod therapy.

As shown in Figure 6, the cumulative proportion of patients free from any disease activity was 41.9%. Multivariate regression analysis revealed lower EDSS score at baseline as the only predictor of disease activity recurrence after fingolimod start (*p* = 0.018).

All patients were discharged at the end of the 6 hours of monitoring, but 8 patients (5.6%) needed 2 hours of extended monitoring due to asymptomatic bradycardia. All these 8 patients were discharged after the extended monitoring. No significant modification of arterial pressure values was also registered.

During the follow-up no serious adverse events were reported. However, headache was reported by 25 patients (17.6%), diarrhea by 7 patients (4.9), and arterial hypertension by 3 patients (2.1%).

Therapy was stopped in 13 patients (9.1%). In 7 patients (4.9%) the interruption was due to lack of efficacy, in 2 patients (1.4%) it was due to general intolerance, and in 3 patients (2.1%) it was stopped due to the patient's decision, and one patient (0.7%) was lost to follow-up.

## 4. Discussion

In this prospective multicenter clinical practice study, fingolimod treatment was effective in providing freedom from disease activity and preventing clinical relapse in patients with RRMS. After starting fingolimod, the cumulative proportion of patients free from clinical relapse throughout the follow-up was 88.1%, and 69.0% of patients remained free from disability progression. Disability progression rates curves disclosed a trend toward further improvement over time. Cumulatively during the study period, almost half of patients were free from any disease activity, that is, no relapses, no disability progression, no new or enlarged T2 lesions, and no gadolinium enhancing lesions.

Of interest, relapse rates were higher in patients with prior natalizumab treatment compared with patients without previous natalizumab exposure (relapse-free survival 77.9% versus 90.5%, resp.). Although prior receipt of natalizumab was associated with a shorter time to first relapse, there was no difference in time to disability progression between patients who had or had not undergone prior natalizumab treatment. It is a widely held notion that natalizumab withdrawal is associated with possible severe disease reactivation occurring within the first three or up to seven months after natalizumab discontinuation. Rather than rebound activity, early relapses are likely to be underpinned by the clearance of natalizumab biological effect prompting recurrence of disease activity. Hence, higher relapse rate and new gadolinium enhancing lesions at 6 months after switching from natalizumab to fingolimod could be possibly driven by early disease recrudescence of disease activity. Recent studies suggest that a wash-out period as short as 4 to 4 weeks should be preferable in order to prevent severe disease reactivation after switching from natalizumab to fingolimod [15]. Nevertheless, our population underwent a three-month wash-out period as required by regulatory agencies in order to prevent potential safety issues from overlapping immunomodulatory effects.

While no direct comparisons are intended, it is of interest to review the findings observed in phase 3 clinical trials of fingolimod and natalizumab in patients with RRMS. The pivotal phase 3 clinical trials, FREEDOMS [7] and TRANSFORMS [9], each of which enrolled over 1000 patients, showed that fingolimod at doses of 0.5 mg and 1.25 mg once daily significantly reduced ARR and MRI disease activity compared with placebo (FREEDOMS) and interferon beta-1a (TRANSFORMS) in patients with RRMS. In the 24-month FREEDOMS study, fingolimod also slowed disability progression, whereas no significant reductions in disability progression had emerged during the 12 months of the TRANSFORMS study. During the 24-month period of the FREEDOMS study, fingolimod reduced the risk of disability progression confirmed at three months (17.7% fingolimod 0.5 mg; 16.6% fingolimod 1.25 mg; 24.1% placebo) [7]. Both fingolimod doses also showed a reduction in the aggregate annualized relapse rate (0.18 fingolimod 0.5 mg, 0.16 fingolimod 1.25 mg) versus placebo (0.40), representing relative reductions in annualized relapse rate of 54% and 60%, respectively.

In the TRANSFORMS study the annualized relapse rate was 0.16 in the 0.5 mg fingolimod group and 0.20 mg in the 1.25 mg fingolimod group versus 0.33 in the interferon group [9].

In the pivotal AFFIRM phase 3 trial of intravenous natalizumab, the 2-year relapse-free rate was 72.41% in the natalizumab group, compared with 46.35% with placebo [16]. In a clinical practice setting, results from 343 consecutive patients with RRMS who started treatment with natalizumab at 12 Italian multiple sclerosis centers have recently been reported [17]. Over 42 months of follow-up, the cumulative proportion of patients free from relapse was 68%, 93% were free from EDSS progression, and 77% were free from MRI activity. Fifty-three percent were free from any disease activity. There was a reduction of ARR of approximately 90% over the follow-up period, compared with the year preceding the start of natalizumab treatment.

Meanwhile, in a recent observational cohort study of 427 RRMS patients receiving either fingolimod or natalizumab, the risk of disability progression was lower in the fingolimod versus natalizumab group (87.39% versus 82.7%, resp.) and a greater proportion of patients receiving fingolimod was relapse-free at 12 months (75.79% fingolimod versus 71.73% natalizumab) [14]. The relapse rate was substantially reduced in both treatment groups within three months of commencing treatment and remained at similar low levels in each group throughout follow-up. Similarly, EDSS was unchanged or improved in similar proportions of both groups and remained stable over 12 months of follow-up.

Treatment with fingolimod was well tolerated, and no patients had any severe adverse events. However, headache was the most common adverse event reported (17.6%). During the study, no concerns with cardiac function were encountered, and no patients had clinically significant bradycardia.

The main focus of our study was to provide real world efficacy data from patients representative of the Italian relapsing-remitting multiple sclerosis population meeting the eligibility criteria of the AIFA for treatment with fingolimod. Although direct comparisons cannot be made, our results in a real world clinical practice setting are to great extent in line with those observed in large clinical trials.

Our findings yield further information to the growing body of evidence for the use of fingolimod in multiple sclerosis in Europe. A recent review article summarized data on the efficacy and safety of fingolimod in patients eligible for therapy according to the European Union label to provide practical guidance on the use of fingolimod in the clinic [18]. In addition to pooled data from clinical trials, the review examined findings from recent European studies of real-world patient populations and concluded that, unlike some newer oral therapies for multiple sclerosis, fingolimod has a well-established safety and efficacy profile. Until additional long-term data on fingolimod are available, clinicians should implement appropriate routine patient monitoring measures to guide and optimize treatment benefits and to minimize adverse events.

We acknowledge that patients enrolled in our post-marketing study were quite heterogeneous in terms of follow-up length, ranging from 1 to 33 months; nevertheless mean follow-up was 14.95 months, and follow-up data were available for at least 57% and 23.9% of patients at 12 and 24 months, respectively. We decided to analyze data of all enrolled patients since literature data indicated an early action of fingolimod due to its mechanism of action [1–5]. Moreover, we included patients starting fingolimod after treatment with natalizumab who are at high risk of relapse in the first months of therapy. Thus, patients with few months of follow-up were also comparable.

We also acknowledge that data from patients with prior natalizumab treatment might have yielded mixed results in regard to previous disease activity and duration. Yet, the aim of our investigation was to perform a clinical study in real world setting as to provide efficacy data from a sample of patients representative of the overall population treated with fingolimod.

## 5. Conclusions

In our study, fingolimod positively influenced the course of RRMS in terms of degree of reduction of relapse, stabilization of EDSS, and reduction of MRI activity. Our data, showing no disease activity in about 50% of patients, confirmed the results of clinical trials.

## Figures and Tables

**Table 1:** Patient demographics and baseline clinical characteristics.

	Total (*n* = 142)	Men (*n* = 47)	Women (*n* = 95)	*p* value
Age (y)	39.56 ± 9.11	39.63 ± 8.15	39.53 ± 9.59	0.954
Duration of disease (y)	11.37 ± 6.74	11.01 ± 6.13	10.62 ± 7.44	0.759
Number of previous DMT	1.51 ± 0.83	1.47 ± 0.80	1.54 ± 0.85	0.644
Duration of DMT (months)	59.52 ± 45.49	32.02 ± 46.45	58.28 ± 45.21	0.647
Previous immunosuppressant therapy	19.01%	25.53%	15.78%	0.164
Previous use of natalizumab	19.71%	12.76%	23.15%	0.143

In the year prior to commencing fingolimod				

EDSS score	2.52 ± 1.02	2.36 ± 1.02	2.60 ± 1.08	0.222
ARR	1.14 ± 0.71	1.06 ± 0.60	1.18 ± 0.78	0.373
New or newly enlarging T2 lesions	72.3%	70.2%	73.4%	0.690
Gadolinium enhancing lesions	49.6%	44.7%	52.1%	0.404

Data are mean ± SD unless otherwise indicated.

ARR: annualized relapse rate.

DMT: disease modifying therapy.

EDSS: Expanded Disability Status Scale.

## Abbreviations

AIFA: Italian Drug Agency
ARR: Annualized relapse rate
CI: Confidence interval
CNS: Central nervous system
EDSS: Expanded Disability Status Scale
EMA: European Medicines Agency
FREEDOMS: FTY720 Research Evaluating Effects of Daily Oral Therapy in Multiple Sclerosis
MRI: Magnetic resonance imaging
MS: Multiple sclerosis
RRMS: Relapsing-remitting multiple sclerosis
S1PR: Sphingosine-1-phosphate receptor
TRANSFORMS: Trial Assessing Injectable Interferon versus FTY720 Oral in Relapsing-Remitting Multiple Sclerosis.
